# Intracoronary imaging for left main percutaneous coronary intervention: a clinical consensus statement of the European Association of Percutaneous Cardiovascular Interventions (EAPCI) of the ESC and the European Bifurcation Club (EBC)

**DOI:** 10.1093/eurheartj/ehag353

**Published:** 2026-05-15

**Authors:** Thomas W Johnson, Nieves Gonzalo, Jose M de la Torre Hernandez, Nicolas Amabile, Peter O’Kane, Mirvat Alasnag, Robert-Jan van Geuns, Niels Ramsing Holm, Salvatore Brugaletta, Lorenz Raber, Remo Albiero, Miroslaw Ferenc, Manuel Pan, Valeria Paradies, Ziad A Ali, Haibo Jia, Pedro Lemos, Rony Mathew, Jun Jie Zhang, Joo-Yoong Hahn, Yiannis S Chiatzizisis, Francesco Burzotta, Adrian P Banning, Giulio Guagliumi, David Hildick-Smith, Alaide Chieffo, Takashi Akasaka, Gary Mintz, Yves Louvard, Jens Flensted Lassen, Goran Stankovic

**Affiliations:** Translational Health Sciences, University of Bristol, Level 7 Queen’s Building, Bristol Royal Infirmary, Upper Maudlin Street, Bristol BS2 8HW, UK; Bristol Heart Institute, University Hospitals Bristol NHSFT, Bristol BS2 8HW, UK; Hospital Clinico San Carlos, IdISSC, Madrid, Spain; Hospital Universitario Marques de Valdecilla, IDIVAL, Santander, Spain; Institut Cardiovasculaire Paris Sud, Paris, France; Department of Cardiology, University Hospitals of Dorset NHS Foundation Trust, Bournemouth, UK; King Fahd Armed Forces Hospital, Jeddah, Saudi Arabia; Department of Cardiology, Radboud University Medical Center, Nijmegen, The Netherlands; Aarhus University Hospital, Aarhus, Denmark; Hospital Clínic, Cardiovascular Clinic Institute, Institut d’Investigacions Biomèdiques August Pi i Sunyer (IDIBAPS), University of Barcelona; Barcelona, Spain; Department of Cardiology, Bern University Hospital, University of Bern, Bern, Switzerland; Interventional Cardiology Unit, Sondrio General Hospital, Sondrio, Italy; University Heart Centre Feiburg, Bad Krozingen, Germany; Department of Cardiology, Reina Sofia Hospital, University of Cordoba (IMIBIC), CIBERCV Cordoba, Bad Krozingen, Spain; Maasstad Hospital, Rotterdam, The Netherlands; Clinical Trials Center, Cardiovascular Research Foundation, NewYork, NY, USA; Department of Cardiology, St.Francis Hospital, Roslyn, NY, USA; NewYork Institute of Technology, Old Westbury, NY, USA; The Second Affiliated Hospital of Harbin Medical University, Harbin, China; Albert Einstein Israelite Hospital, Sao Paulo, Brazil; Lisie Heart Institute, Lisie Hospital, Kochi, India; Nanjing First Hospital, Nanjing Medical University, Nanjing, China; Samsung Medical Center, Sungkyunkwan University School of Medicine, Seoul, South Korea; Division of Cardiovascular Medicine, Leonard M. Miller School of Medicine, University of Miami, Miami, FL, USA; IRCCS Foundation Agostino Gemelli University Hospital, Rome, Italy; John Radcliffe Hospital, Oxford University Hospitals, Oxford, UK; IRCCS Galeazzi Sant’Ambrogio Hospital, Milan, Italy; Sussex Cardiac Centre, Royal Sussex County Hospital, Brighton, UK; Vita Salute San Raffaelee University, San Raffaele Scientific Institute, Milan, Italy; Nishinomiya Watanabe Cardiovascular Cerebral Center, Hyogo, Japan; Cardiovascular Research Foundation, NewYork, NY, USA; Institut Cardiovasculaire Paris Sud, Paris, France; Odense University Hospital, Odense, Denmark; Department of Cardiology, Clinical Center of Serbia, and Faculty of Medicine, University of Belgrade, Belgrade, Serbia

**Keywords:** Percutaneous coronary intervention, Intracoronary imaging, Optical coherence tomography, Intravascular ultrasound, Bifurcation, Left main stem

## Abstract

Left main disease represents one of the most complex lesion subsets for percutaneous coronary intervention, associated with an increased risk of serious late complications. The recent endorsement of intracoronary imaging for the guidance of left main bifurcation percutaneous coronary intervention in both acute and chronic coronary syndrome, with respective recommendations in American and European guidelines, reflects the results of recent studies. Patient benefit can only be realized through the interventional community embracing the need for intracoronary imaging-guided planning, guidance and optimization of left main stenting.

This clinical consensus statement summarizes the views of a global expert panel, coordinated by the European Association of Percutaneous Cardiovascular Interventions (EAPCI), in collaboration with the European Bifurcation Club (EBC). The document includes an appraisal of the most contemporary evidence and provides clinical guidance on how to maximize the benefit of intravascular ultrasound or optical coherence tomography in the treatment of left main bifurcation disease.

## Graphical Abstract

**Graphical Abstract:**
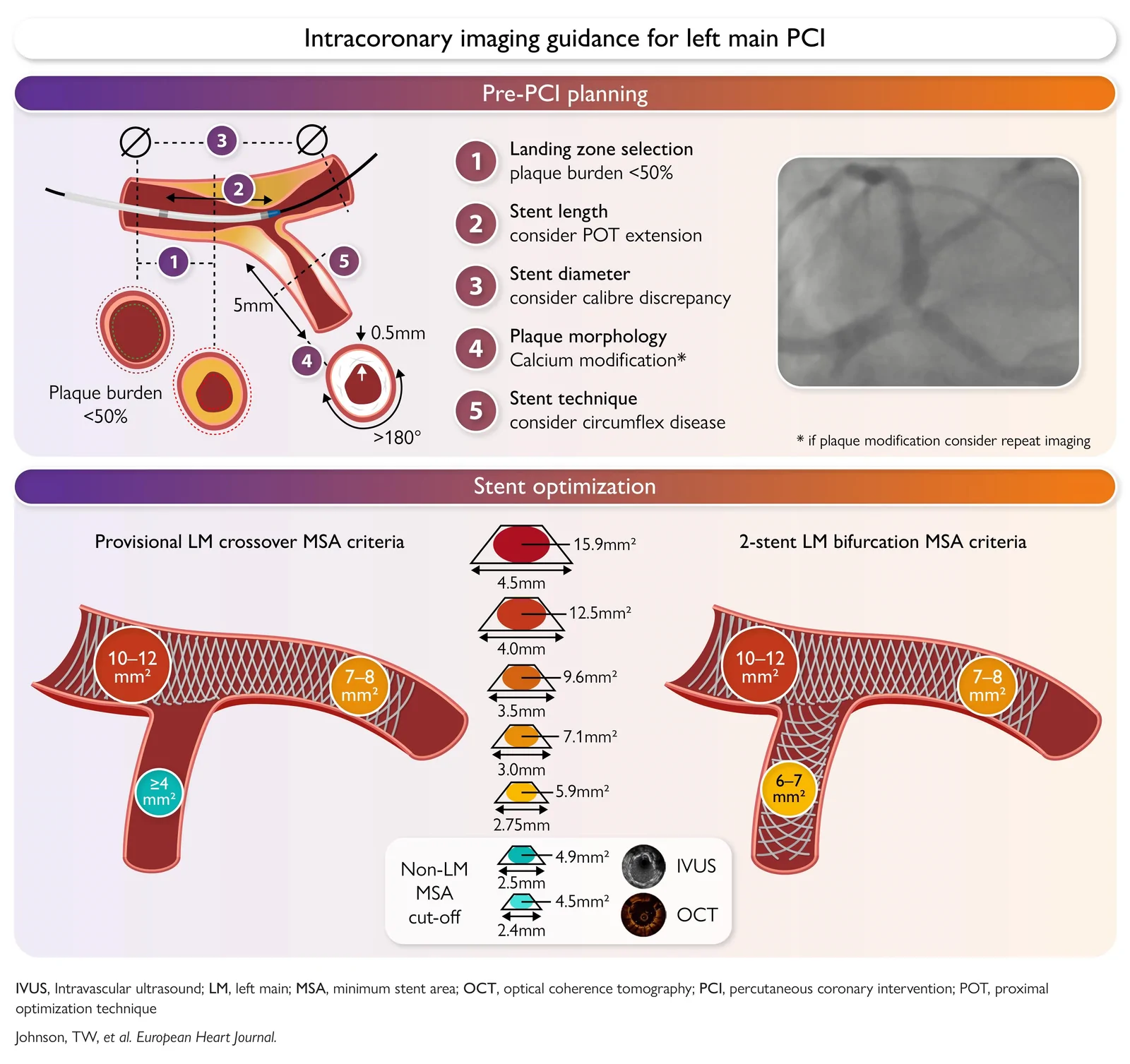
Pre and post-PCI criteria for the effective guidance of left main stenting using intracoronary imaging.

## Introduction

Recent guidelines have identified left main coronary artery (LM) disease as a complex lesion type that benefits from intracoronary imaging (ICI) guidance to minimize future adverse clinical outcomes.^1–3^ However, European adoption of ICI remains low, in comparison with East Asian colleagues,^4^ despite an increased awareness that LM treatment is complex and when not optimized, associated with poor clinical outcomes.^5^

The European recommendation for the use of ICI in LM treatment is welcomed by the European Association of Percutaneous Cardiovascular Interventions (EAPCI) and European Bifurcation Club (EBC).^1^ However, this recommendation must be taken in the context of coronary artery bypass grafting (CABG) being acknowledged as the overall preferred mode of revascularization (Class I, level of evidence A). The guidelines support revascularization by percutaneous coronary intervention (PCI) in patients with significant LM stenosis of low complexity (SYNTAX score ≤22) (Class I, level of evidence A) or in more complex patients, when considered not suitable for CABG (Class IIB, level of evidence B). In the light of this shift in recommendation from the previous endorsement of LM PCI,^6^ with greater emphasis on PCI needing to provide equivalent completeness of revascularization to that of CABG, we find it is critically important that our community embraces best practices and enhances their understanding of how to utilize ICI for the guidance and optimization of LM PCI.

This clinical consensus statement intends to build upon previous documents that have focused on the role of intravascular ultrasound (IVUS) and optical coherence tomography (OCT) in LM and bifurcation PCI,^7–9^ incorporating the latest evidence and providing colleagues with a systematic approach to the use of ICI in guiding their procedures from lesion assessment, through procedural planning and optimization of the acute stenting result.

## Existing data with comparison of intravascular ultrasound vs optical coherence tomography

Coronary angiography has been used to guide PCI since its inception. However, angiography's limitations, including low spatial resolution, frequent overlapping branches with failure to provide true orthogonal projections of the bifurcation limiting the ability to diagnose either stent underexpansion or inadequate lesion coverage, are increased during LM PCI. Furthermore, the LM bifurcation is more frequently associated with calcium, treatment with multiple, potentially overlapping or interdigitated stent segments, as well as a higher risk of unintended stent deformation, all factors that increase the risk of a suboptimal PCI result.

ICI using either IVUS or OCT provides high-resolution images of the coronary artery and a comprehensive assessment of lesion pathology and subsequent stent deployment. Importantly, the two modalities have differing strengths and weaknesses for use in guiding LM PCI, which will be explored later in the document (*Figure 1*). The choice of ICI modality should primarily be driven by the familiarity of the operator/institutional team in the interpretation of ICI data and the specific characteristics of the patient and/or lesion (e.g. advanced chronic kidney disease and/or ostial LM lesion extension benefiting from the use of IVUS).

**Figure 1:**
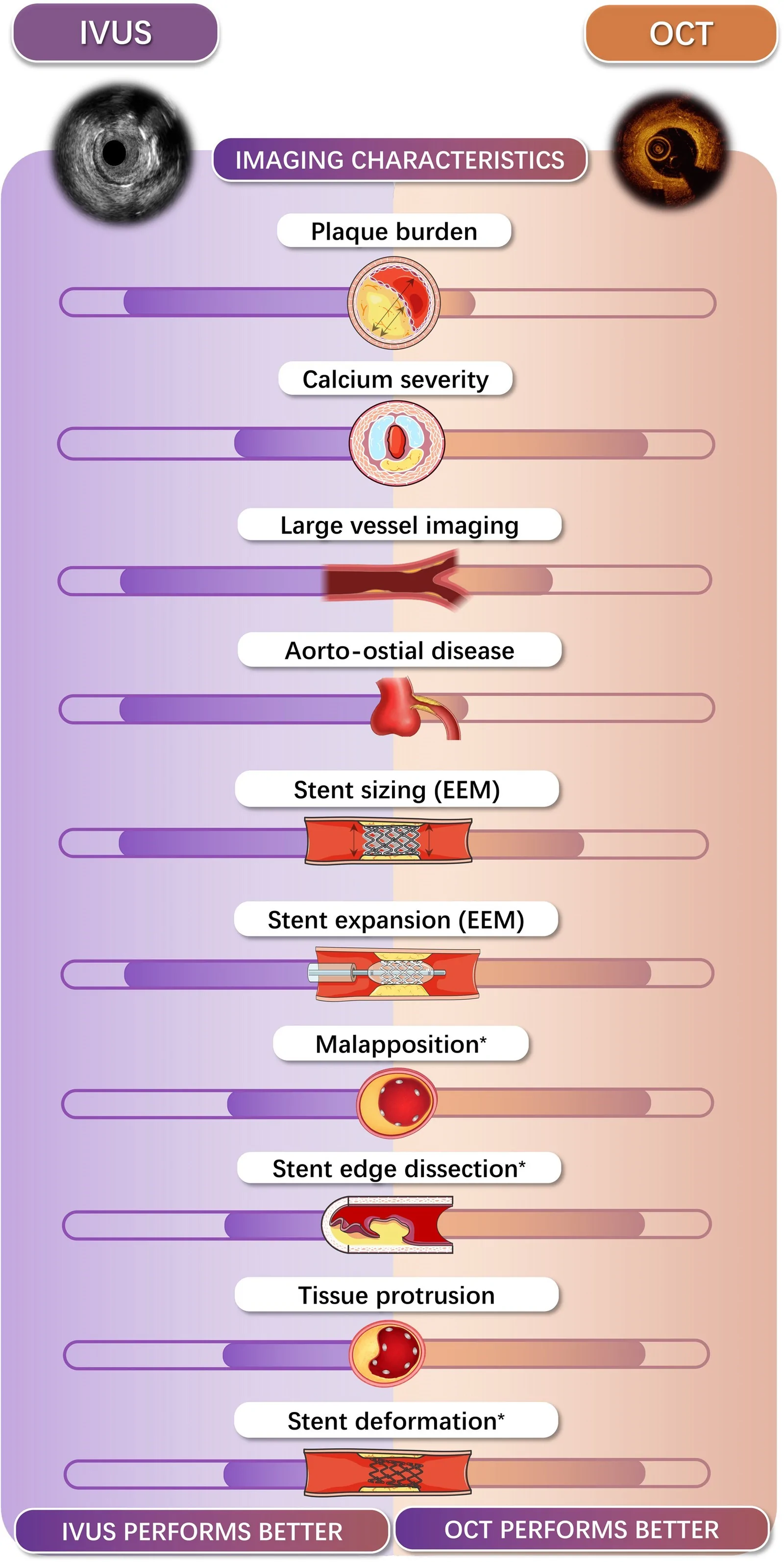
A graphical comparison of the capabilities of IVUS and OCT in guiding LM PCI (*where LM stent extends to ostium, proximal visualization superior with IVUS)

IVUS has been used clinically for over 25 years; and consequently is more frequently used, as evidenced by the 3:1 use, compared with OCT, observed in the Korean RENOVATE-COMPLEX-PCI study.^10^ IVUS is often preferred for LM PCI due to providing a larger field of view and consistent assessment of the ostial LM segment, as observed in EBC MAIN, where 39% of cases used intravascular imaging with 84% of these cases preferring IVUS.^11^ The introduction of high-definition IVUS has provided enhanced axial resolution (30–40 µm) which may be preferred against conventional IVUS catheters in the management of LM PCI. However, OCT provides higher resolution (10–20 µm), with contemporary devices offering three-dimensional (3D) capabilities that are well suited to bifurcation assessment, pre- and post-stenting. The LEMON study confirmed the feasibility of using a standardized OCT protocol to guide distal LM PCI.^12^ Furthermore, the recent OCTIVUS trial directly compared IVUS- and OCT-guided PCI^13^; within the trial, there were 264 patients with LM disease requiring treatment, and the trial demonstrated equivalent outcomes in the overall population and the predefined subgroup of LM patients. Therefore, we support the use of IVUS or OCT in LM PCI and suggest operators select the device they have the greatest confidence in interpreting or consider best suited to the patient/lesion anatomy/procedural characteristics.

Demonstrating a truly meaningful and cost-effective benefit from ICI during PCI has always been challenging. It is presumed that most benefit will be evident in more complex PCI, and LM PCI is recognized as among the most challenging lesion subsets. Non-randomized retrospective case series have suggested a clinical benefit from adjunctive IVUS during LM PCI and are summarized within the EBC left main imaging consensus,^8^ however, it is important to recognize that selection bias, favouring the use of ICI in stable/lower risk patients, may have influenced these findings. These non-randomized data are augmented by three small randomized clinical trials (RCT),^14–16^ a pre-specified subanalysis of a large, prospective study of LM PCI (ROLEX),^17^ the LM sub-study of RENOVATE-COMPLEX-PCI,^18^ and real-world national data such as the British Cardiovascular Intervention Society (BCIS) registry reporting that the use of IVUS resulted in a reduction in events and especially patient mortality.^19^

In contrast to this accumulation of data, favouring an IVUS-guided approach to LM treatment, the first dedicated RCT to compare IVUS- vs angiography-guided PCI for unprotected LM disease, OPTIMAL, has recently demonstrated no benefit from an imaging-guided approach, with respect to the incidence of stroke, myocardial infarction, any revascularization, or death from any cause at a median follow-up of 2.9 years.^20^ The study population was complex, with a mean SYNTAX score of 29.7 ± 12.6 and event rates were higher than anticipated. The use of IVUS was strongly recommended pre and post-stenting, with pre-PCI imaging undertaken in 65.1% (261/401 patients), and post-stenting IVUS triggered an optimization step in 29.3% (111/379 patients), most commonly further post-dilatation. Site reported post-procedural IVUS findings demonstrated an LM MSA of 11.62 ± 3.70 mm^2^, LAD MSA of 8.02 ± 2.56 mm^2^ and Cx MSA of 6.71 ± 2.17 mm^2^.

Three large studies have assessed the impact of OCT on PCI outcomes. In ILUMIEN IV,^21^ left main PCI was excluded. In OCTOBER,^22^ patients with LM disease comprised 19% of the study population, and although results for LM were not reaching statistical significance, in view of the size of the LM subgroup, the point estimates were directionally consistent with the overall treatment group (*Figure 2*). In addition, the effects were diluted by the study protocol allowing the use of IVUS in the angiography arm of the LM subgroup, with IVUS use in 35.3% of the angio-guided LM PCIs. In OCCUPI,^23^ LM disease comprised 14.3% of patients, and in line with the primary outcome of the study, OCT-guided PCI was associated with lower 1-year MACE compared with angiography guidance (*Figure 2*).

**Figure 2:**
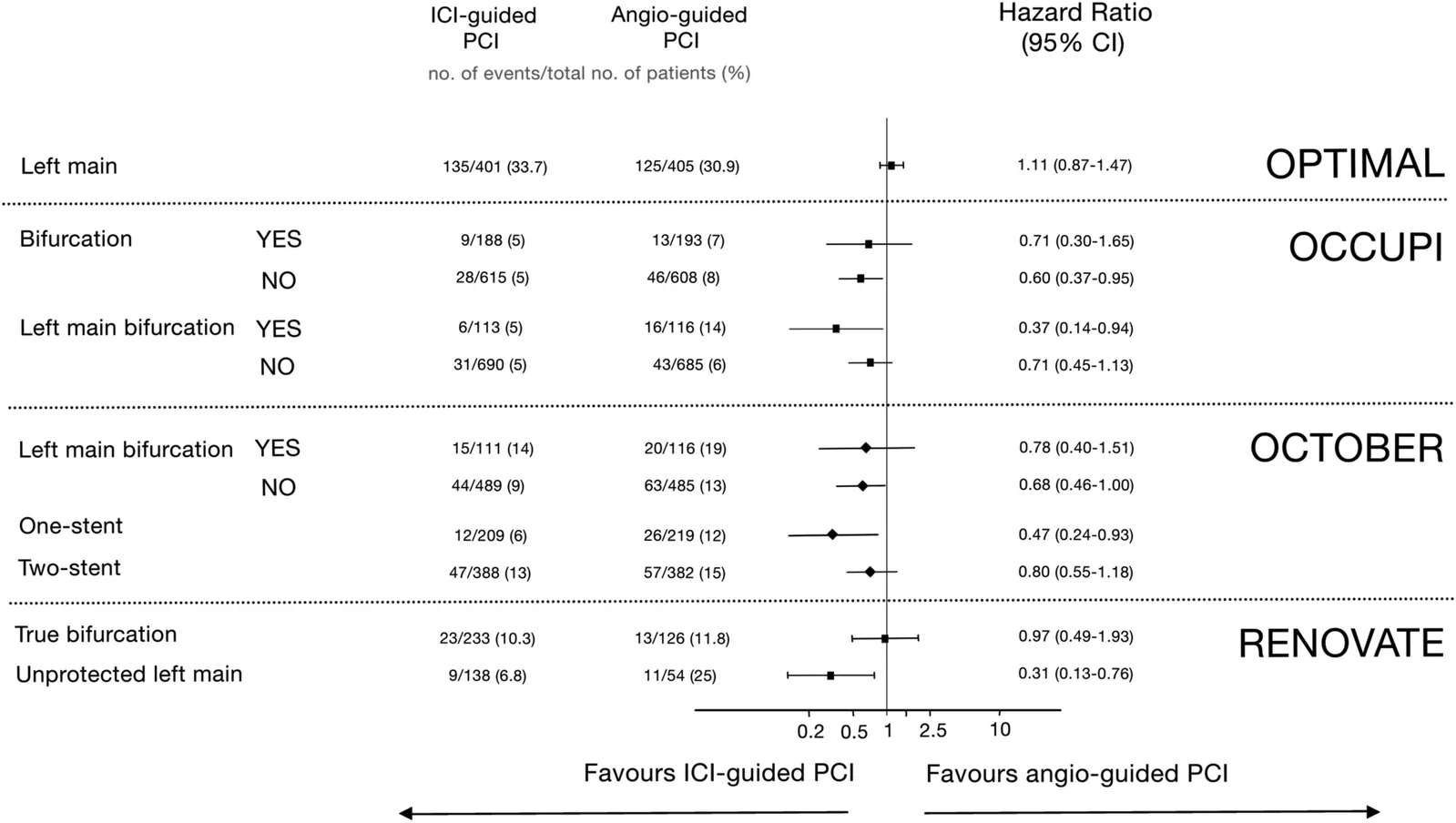
A composite Forrest plot of contemporary intracoronary imaging studies OPTIMAL,^20^ OCCUPI,^23^ OCTOBER^22^ & RENOVATE,^10^ assessing the impact of ICI on bifurcation PCI

After the publication of RENOVATE-COMPLEX-PCI, ILUMIEN IV, and OCTOBER, an updated meta-analysis suggested reductions in the rates of cardiac and all-cause mortality after imaging-guided LM PCI.^24^ A dedicated bifurcation meta-analysis has demonstrated ICI guidance reduces the risk of target vessel failure compared with angiographic guidance.^25^

Notwithstanding the results of OPTIMAL, it is interesting to observe that sub-group analysis of the most contemporary studies has failed to demonstrate a benefit of ICI in true bifurcation lesions, but when restricted to LM, an advantage of ICI guidance was observed^10, 23^ (*Table 1* and *Figure 2*). This difference in outcome likely reflects the relative consistency of anatomy/approach to the LM, compared with smaller calibre non-LM bifurcations.

**Table 1:** Overview of contemporary studies testing the role of intra-coronary imaging guidance for left main percutaneous coronary intervention

Study name	Years of recruitment	*N* (ICI/Whole cohort)	ICI cohort		Primary endpoint	ICI events		Angio events	HR (95% CI)	*P*-value
			IVUS	OCT/OFDI		IVUS	OCT/OFDI			
NOBLE LM substudy^26^	2008–2015	435^a^/603	435 (100%)	-	**5-year** MACCE (all-cause death, non-procedural MI, repeat revascularization, or stroke)	82 (18.9%)	-	41 (25.0%)	0.70 (0.49–1.03)	.07
ROCK II^27^	2012–2017	377/730	215 (57%)	162 (43%)	**1-year** TLF (cardiac death, TV-MI, or TLR)	29 (13.5%)	19 (11.7%)	75 (21.2%)	**IVUS vs Angio** 0.63 (0.42–0.94) **OCT vs Angio** 0.55 (0.34–0.88)	.02 .01
EBC Main^11^	2016–2019	179/455	151 (84%)	28 (16%)	**1-year** composite (all-cause death, MI, or TVR)	30 (16.8%)		43 (15.6%)	0.94 (0.55–1.59)	.812
ROLEX-LM^17^	2017–2020	200/450	188 (94%)	12 (6%)	**1-year** TLF (cardiac death, TV-MI, or ischaemia-driven revascularization)	2.0%^b^		7.6%	0.28 (0.13–0.58)	<.001
OCTOBER LM subgroup^22^	2017–2022	111/227	-	111 (100%)	**2-year** MACE (cardiac death, TL-MI, or ID-TLR)	-	15 (13.5%)	20 (17.2%)^c^	0.78 (0.40–1.51)	-
LEMON^12^	2018–2019	70/70	-	70 (100%)	**1-year** MACE (cardiac death, stent thrombosis, TVR)	-	1 (1.4%)	-	-	-
RENOVATE LM substudy^18^	2018–2021	138/192	128 (93%)	10 (7%)	**3-year** TVF Kaplan-Meier event rate (cardiac death, TV-MI, or clinically driven TVR)	6.8%		25.1%	0.31 (0.13–0.76)	.010
OCCUPI (LM subgroup)^23^	2019–2022	113/229	-	113 (100%)	**1-year** MACE (cardiac death, MI, stent thrombosis or ID-TVR)	-	6 (5.3%)	16 (13.8%)	0.37 (0.14–0.94)	-
OPTIMAL^20^	2020–2023	401/806	401 (100%)	-	POCE at **median 2.9 years** (any stroke, MI, revascularization, or all-cause death)	135 (33.7%)	-	125 (30.9%)	1.11 (0.87–1.42)	.40

HR, hazard ratio; CI, confidence interval; ICI, intracoronary imaging; IVUS, intravascular ultrasound; MI, myocardial infarction; OCT, optical coherence tomography; OFDI, optical frequency domain imaging; MACCE, major adverse cardiovascular and cerebrovascular events; TLF, target lesion failure; TV-MI, target vessel myocardial infarction; TLR, target lesion revascularization; TVR, target vessel revascularization; MACE, major adverse cardiac events; TL-MI, target lesion myocardial infarction; ID-TVR, ischaemia-driven target vessel revascularization; POCE, patient-oriented composite endpoint.

^a^Post-PCI IVUS used.

^b^Primary endpoint evaluated using cumulative incidence functions; with covariate balance propensity score estimation.

^c^41/116 angio cases used IVUS (35.3%).

Further analyses of the most contemporary studies are required, and consideration of the true utility of ICI is needed to fully understand the impact of these technologies on patient outcomes. It remains important to highlight the dual challenge of undertaking complex stenting techniques and interpreting imaging in true bifurcation lesions. We believe that a standardized approach in the use of ICI to guide treatment of complex bifurcation lesions is needed.

### Pre-percutaneous coronary intervention imaging assessment

Assessment of LM-lesions before intervention provides an operator with numerous advantages, including an alternative assessment of lesion significance, delineation of plaque morphology, with the potential to select modification techniques to ensure optimal stent expansion, an understanding of disease extension across all segments of the bifurcation, possibly impacting the choice of stenting technique and ultimately accurate sizing of stents for treatment.

Despite the obvious utility of pre-PCI ICI adoption, it must be acknowledged that the proximal nature of LM lesions increases the risk of haemodynamic instability, so care must be taken to ensure safe image acquisition, often achieved through pre-dilatation of a significant lesion.

#### Lesion significance

The interpretation of LM stenosis significance by angiography is particularly challenging. Given that the mass of myocardium supplied by the LM does not have as much variability as other areas of the coronary tree, several studies have attempted to find an intravascular imaging-derived minimal lumen area (MLA) cut-off to determine LM severity, reporting values between 4.5 and 8.0 mm^2^ (*Table 2*).^28–32^ It is important that pullbacks are obtained from both left anterior descending artery (LAD) and left circumflex artery (LCx) into the LM as the MLA, estimated from one vessel, may be inaccurately oversized due to oblique imaging probe position (*Figure 3*). The smallest MLA value obtained from either of the two vessel pullbacks should be used for clinical decision-making. Furthermore, we advocate the use of an automated pullback, at low speed (0.5–1 mm/s) to ensure uniform assessment of the LM segment, assuming that the patient is haemodynamically stable.

**Figure 3:**
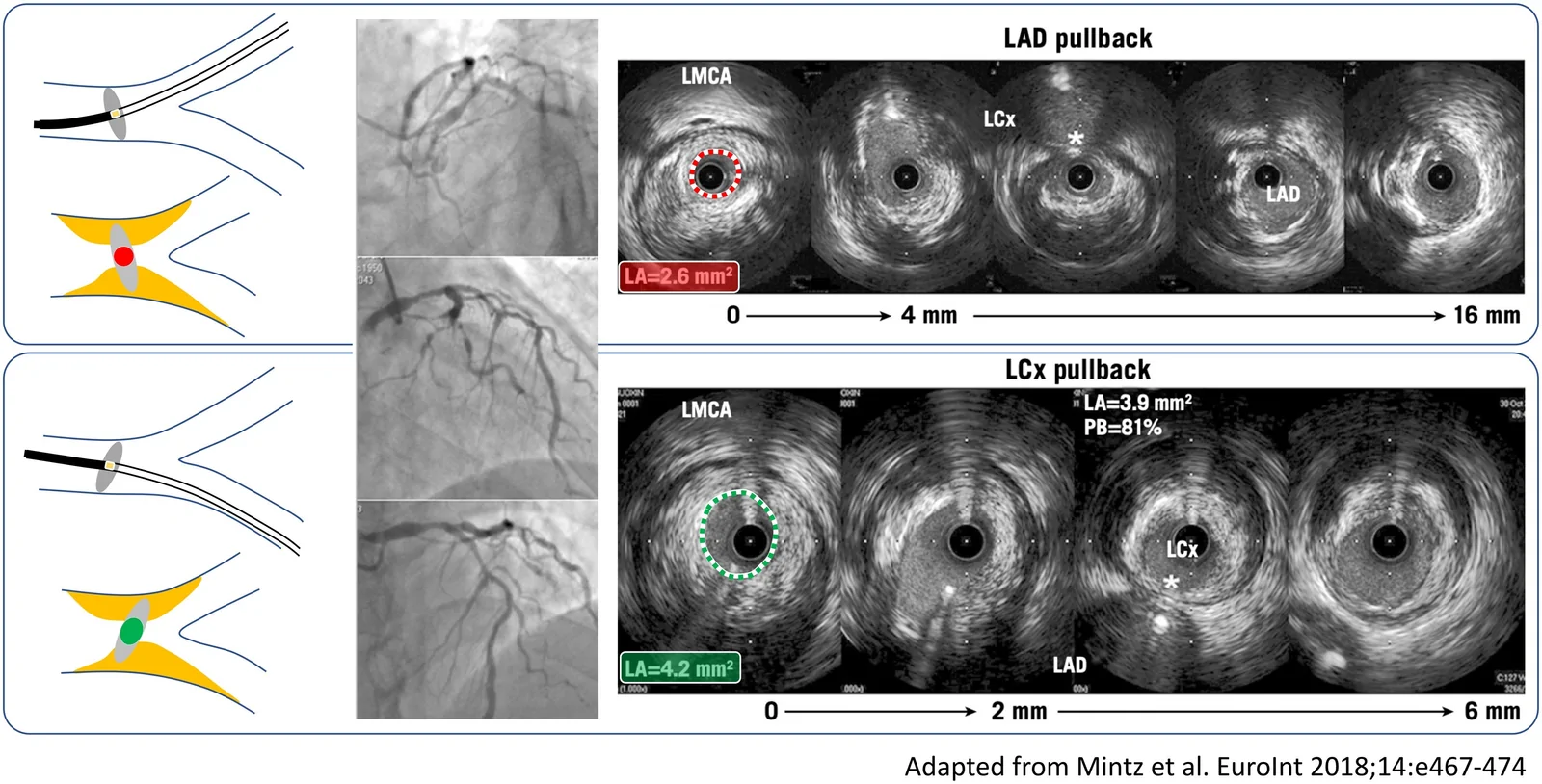
Intravascular-ultrasound guided assessment of left main disease significance—the impact of catheter alignment on minimal lumen area measurement. Complete assessment requires IVUS evaluation of both limbs with the lowest value taken to determine significance. This case example, adapted from the EBC IVUS left main consensus,^8^ demonstrates significant differences in the lumen area between LAD and circumflex (LCx) pullback with the most severe MLA recorded from the LAD (red dot and dotted line)

**Table 2:** Proposed intravascular imaging derived cut-off values for minimal lumen area in intermediate left main stenosis

Study	Patient number	Method	Cut-off MLA
IVUS			
Legutko *et al*.^32^	44	IVUS vs FFR <0.75	8 mm^2^
Jasti *et al*.^28^	55	IVUS vs FFR <0.75	5.9 mm^2^
Fassa *et al*.^29^	214	Population-based normal range	7.5 mm^2^
de la Torre Hernandez *et al*.^30^	354	Prospective clinical validation	6 mm^2^
Park *et al*.^33^	112	IVUS vs FFR <0.8	4.5 mm^2^
OCT			
Bouki *et al*.^31^	101	OCT vs FFR <0.8	5.4 mm^2^

FFR, fractional flow reserve; IVUS, intravascular ultrasound; MLA, minimal lumen area; OCT, optical coherence tomography.

The IVUS-derived MLA cut-off of 6 mm^2^ is most frequently cited and is supported by a correlation study with pressure wire (using a fractional flow reserve [FFR] threshold of 0.75),^28^ application of linear law in keeping with the fractal nature of the vasculature,^34^ and prospective clinical validation in the multicentre LITRO study.^30^ However, the Part 2 EAPCI consensus statement on ICI^35^ suggested a threshold of 4.5 mm^2^, acknowledging the data derived from an Asian population (using an FFR threshold of 0.80)^33^ and the challenge of using an absolute cut-off regardless of patient size (*Table 2*). There are concerns that reducing the absolute cut-off value impacts unfavourably on the sensitivity of deferral.^36^ Consequently, it was proposed that MLA values between 4.5–6 mm^2^ should lead operators to consider invasive physiology assessments. ICI- and angiography-derived physiological assessment is rapidly advancing but at present lacks robust data for application in the evaluation of LM disease.^37,38^

We propose that additional clinical characteristics and plaque morphological features may further influence the decision to proceed to revascularization. ICI features of high-risk plaque, including plaque disruption, with or without luminal thrombus, or intra-plaque haemorrhage, may support a decision to revascularize. Therefore, invasive physiology, ICI features, and/or patient characteristics may influence the decision to revascularize when encountering an LM stem MLA of 4.5–6 mm^2^ (*Figure 4*).

**Figure 4:**
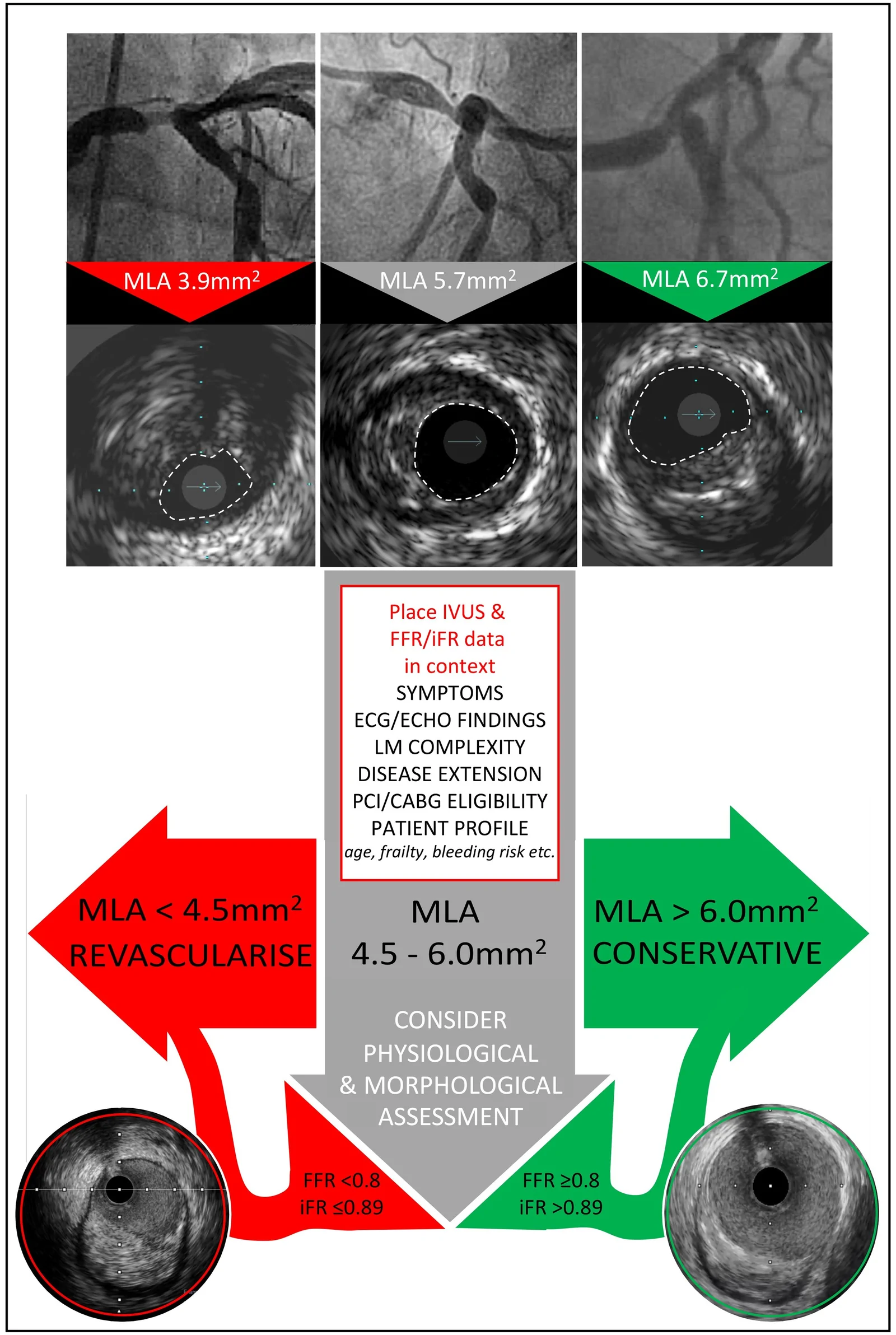
Intravascular-ultrasound guided assessment of left main disease significance—absolute minimal lumen area thresholds for guiding revascularization. Adapted from the second EAPCI ICI consensus^35^ to include patient and ICI criteria to guide decision-making

Sizing by non-high definition IVUS is known to provide larger measurements than OCT. Consequently, the OCT cut-off value could be ≈10% smaller, as suggested by a correlation study with FFR.^31^ However, an OCT-derived MLA cutoff for deferral of LM PCI has not been formally tested, but one international study is ongoing (OPTICO-LM, NCT03820492) comparing invasive FFR with OCT MLA and FFR from computed tomography (CT). Beyond MLA, OCT can provide information regarding the underlying plaque morphology and evidence of disruption (erosion/rupture/associated thrombus), which may have potential implications for the revascularization decision.^39^

#### Plaque morphology: plaque preparation

Bifurcation lesions are commonly associated with high burden calcium^40^ and subsequently a risk of stent underexpansion.^41^ Both IVUS and OCT more accurately identify presence of calcific disease than angiography.^42^ Quantification of the burden of calcium provides a useful prediction of stent underexpansion and should inform operators of the need to undertake advanced modification.^41^ Qualitative assessment of calcium has identified calcified nodule as particularly prone to unfavourable long-term PCI results,^43,44^ so identification and modification is preferred, especially if ICI confirms advantageous wire bias maximizing contact of a modification device with calcific tissue. Many algorithms for calcium modification exist but there is no robust evidence to support either the selection of one modality according to calcific burden/morphology or which parameters demonstrate adequate lesion preparation. ICI appears fundamental in guiding the treatment of severely calcific coronary disease and an ongoing study is testing the role of a standardized algorithm for plaque modification in de-novo LM and non-LM chronic calcific coronary artery disease (CYCLOPES).^45^

We encourage the use of a quantitative score to predict the risk of stent underexpansion and when to consider the use of adjunctive tools^41,46^ but would also encourage operators to repeat imaging, following lesion preparation, to ensure that sufficient calcium fractures were achieved, allowing maximal lesion expansion before selection and implantation of stents (*Figure 5*). This should be performed in concert with assessment of balloon expansion, where full expansion with a balloon sized 1:1 to the reference segment is achieved.

**Figure 5:**
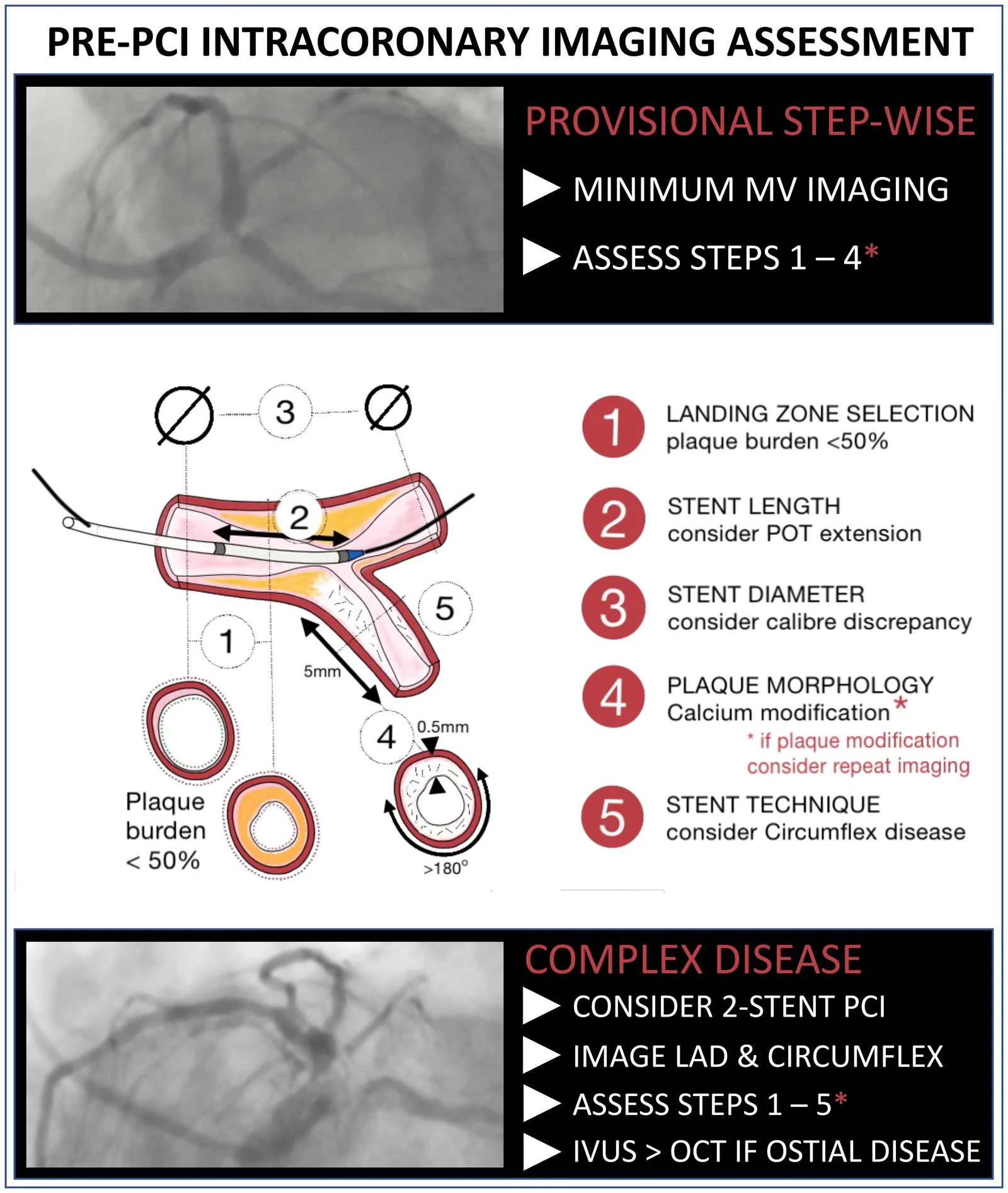
Step-wise approach to maximizing the benefit of pre-PCI ICI. 1. Assessing the landing zones with particular attention to avoidance of plaque burden >50%. 2. Stent length selection should consider the need for proximal extension of the POT balloon to overcome significant calibre discrepancy^3^ between proximal and distal bifurcation segments. 4. Identification of significant calcification, especially arc >180^°^, length >5 mm and thickness >0.5 mm should trigger use of adjunctive technologies and repeated imaging is recommended to ensure adequate preparation before stenting. 5. In the presence of complex bifurcation disease affecting both limbs of the bifurcation, ICI of both the left anterior descending and circumflex arteries may inform procedural decision making

#### Landing zone selection

In the simplest form, plaque identification is needed to define appropriate stent landing zones, ideally selecting vessel segments without significant atherosclerosis (preserved three-layer vessel wall structure by ICI). Since such healthy vessels are often not present in patients with diffuse atherosclerosis, stent landing zones should be segments with <50% plaque burden (IVUS) or where visualization of the external elastic membrane is maximal in the reference segment (OCT). Importantly, ICI identification of high burden plaque extending the length of the LM should guide an operator to complete LM stent coverage.

High-burden lipidic plaque should be avoided for stent landing due to a heightened risk of edge dissection and subsequent restenosis.^47^ Additionally, plaque distribution has been identified as a predictor of side branch (SB) closure. Consequently, greater care should be taken where high-burden lipidic plaque is located at the SB ostium.^48^ Where it is anticipated that the preferred landing zone is in a segment with significant plaque burden, it is advised to base stent selection on lumen rather than vessel dimensions, with additional consideration of bifurcation segment calibre discrepancies requiring significant stent oversizing. Consequently, both quantitative and qualitative lesion assessment, in optimal stent selection, is advised. Similarly, vessel preparation should be determined by the same quantitative and qualitative criteria. Predilatation, sized 0.5 mm less the ICI-defined reference lumen or vessel diameter (dependent upon the presence or absence of significant plaque, respectively), with angiographic assessment for uniform balloon expansion is advised. As discussed previously, where expansion is limited, further imaging to confirm plaque modification, with the use of 1:1 reference vessel:balloon sizing or additional adjunctive device use, such as scoring or cutting balloon may be appropriate. Where significant discrepancy exists between proximal and distal vessel/lumen diameters, then predilatation, appropriately sized to the individual vessel segments, should be undertaken sequentially.

#### Geometry and decision-making regarding stent platform and stent technique

The LM and its atherosclerotic involvement is characterized by a series of features that are expected to strongly influence stent implantation procedures.^49^ Particularly, according to the LM morphology (take-off, length, branching) and plaque distribution (ostial, mid-shaft or distal bifurcation lesions), two different scenarios are commonly faced.

The first (and less common) is represented by the condition where the distal LM is not diseased, so that isolated LM stenting is feasible, assuming sufficient LM length to accommodate a stent without ostial or bifurcation overlap. When these conditions are recognized, the selection of a single drug-eluting stent with appropriate diameter and length is expected to warrant an efficient PCI result. To guide this approach, ICI represents the ideal tool to (i) confirm the presence of a healthy distal landing zone, (ii) accurately assess the LM size, and (iii) evaluate the length of LM to be covered with confirmation of landing sites.

Whilst ensuring complete lesion coverage, stenting of the entire LM may not be necessary and ICI provides confidence in determining the presence of a proximal landing zone. If concerns regarding dissection exist (see Section *Landing zone—dissection management*), then complete LM coverage is desirable, however, where a significant segment of proximal vessel provides healthy landing, avoidance of the ostium may limit risk of malapposition due to an oversized ostial geometry, inadvertent geographical miss with aortic extension and/or associated guide catheter collision and stent deformation. While IVUS can efficiently provide all this information, OCT may be limited by the need to visualize the ostial LM segment, which is commonly obscured by guide catheter or blood contamination^50^ (see Section *Landing zone—dissection management* for an approach to overcome this), which is a relevant consideration when assessing lesion and stent length.

The second (and more common) scenario is represented by variable degrees of atherosclerotic involvement of the LM bifurcation requiring stent implantation from the LM into the LAD/LCx. When selecting a stent to span the distal bifurcation it is critically important to recognize the difference in calibre between proximal and distal main vessel, with consideration of the expansion limits of the stent selected to treat the diseased segment. Disease limited, angiographically, to an ostial daughter vessel (Medina 01,0 or 00,1) poses an additional procedural challenge regarding the decision to extend stenting proximally into the LM or limit treatment to the distal bifurcation segment (‘nailing the ostium’). Previously, IVUS has demonstrated that disease is rarely isolated to either the LAD or circumflex ostium, with extension back into the distal LM observed in more than 90% or 80%, respectively.^51^ This finding highlights the importance of pre-procedural imaging to define the distribution of disease and assist in treatment planning. Therefore, it is generally advised to avoid ostial stent placement due to the risk of plaque disruption, geographical miss, or inadvertent proximal stent protrusion/gross malapposition with a heightened risk of subsequent adverse patient outcomes.^52^ However, there may be scenarios where isolated ostial treatment is deemed preferable, for example, when gross disparity in LM to distal vessel calibre would prevent over-expansion of a proximal stent segment. In such circumstances, ICI-guided ostial stent placement^53^ or a drug-coated balloon strategy^54^ may be appropriate.

LM bifurcation PCI is recognized to associate with a higher risk of failure,^55^ so meticulous pre-procedural planning of the selected bifurcation stenting technique^56^ is pivotal. It is for this reason that the EBC continues to advocate a provisional strategy for the treatment of complex bifurcation lesions, as the technique offers a step-wise approach, aiming to keep the procedure simple but facilitating escalation (culotte or T-and-small protrusion) according to peri-procedural challenges that may not have been anticipated prior to commencement. Upfront treatment of the circumflex, through selection of the double-kissing (DK)-Crush technique, or by inverted provisional (LM-circumflex stenting first followed by proximal optimization technique [POT], kissing and LAD stenting according to T/TAP or culotte) may be appropriate where the operator perceives the anatomy/disease complicates circumflex access or occlusion risk is deemed to be high (see Section *Side branch assessment*). Regardless of strategy adopted, pre-stenting ICI facilitates assessment of the burden, morphology and location of disease, with accurate sizing and length assessments for stenting, where angiography commonly leads to an underestimation of complexity.^51^ Contemporary trials have attempted to standardize the approach to ICI guidance, examples include the IVUS guidance protocol designed for DK-Crush stenting^57^ and the OCT-guidance protocol for stenting in complex bifurcation lesions, successfully adopted in the OCTOBER trial.^58^

#### Side branch assessment

We acknowledge that there must be a balance between complete evaluation of the bifurcation lesion and simplification of the PCI approach, with the provisional step-wise strategy appropriate for the majority of cases. However, perceived complexity of the bifurcation, or angiographic ambiguity, may require consideration of circumflex treatment and therefore ICI of both branches may be advisable pre-PCI (*Figure 5*).

##### Predictors of side branch compromise

Historical data have identified the circumflex ostium as being particularly prone to carinal shift and acute lumen loss in crossover stenting, especially where there is a narrow angle between LAD and circumflex plus a wide angle between LM and circumflex.^59^ In the absence of angiographic features of complexity (circumflex diameter stenosis <50%), a pre-procedural ostial circumflex MLA <3.7 mm^2^ or plaque burden >56% have been shown to be predictive of a post-stenting FFR <0.80 following cross-over provisional stenting.^48^ However, the positive predictive value of these pre-treatment thresholds were poor at predicting post-procedural compromise of the SB.

The Bifurcation Academic Research Consortium detailed angiographic, intravascular imaging and CT coronary angiography criteria for complex bifurcation, with shared criteria including true bifurcation disease (medina 1,1,1, 1,0,1, or 01,1) plus one of the following (SB disease length ≥10 mm, thrombotic lesion, calcium arc >60^°^ at the culprit site or perceived difficulty with SB access (retro-flex circumflex with bifurcation angle between LM and circumflex <90^°^).^60^

Therefore, we would propose a composite assessment of complexity/risk of circumflex (SB) compromise, combining angiographic and ICI features of the ostial and proximal circumflex segments:

1. Angiographic circumflex diameter stenosis ≥70%
2. ICI evaluation of MLA <4 mm^2^ or plaque burden >55%
3. Circumflex lesion length extension ≥10 mm
4. Significant calcification in circumflex (SB)—we would advise that when there is evidence of moderate/severe calcification by angiography, that ICI is used to better define the qualitative and quantitative extent of calcification to guide need for modification. Particular attention given to ICI-defined calcification with arc >180°, >5 mm in length, and >0.5 mm thickness
5. Evidence of plaque instability +/− intra-luminal thrombus

## Post-percutaneous coronary intervention imaging assessment

Bifurcation PCI ICI-guided optimization provides both acute (procedural success) and chronic (long-term clinical outcome) benefits. As already explained, an ICI-guided approach is best achieved through adoption of imaging from the very start of the procedure. However, it is important to recognize that much of the existing data, as outlined in Section *Existing data with comparison of IVUS vs OCT*, precedes the development of pre-specified protocols for ICI guidance and consequently it cannot be assumed that ICI has been used to its full potential. Furthermore, the benefits observed in registries may underestimate the advantage provided by ICI in guiding LM PCI. Consistent with this, de la Torre Hernandez and colleagues suggested that the adoption of a dedicated protocol for IVUS-guided LM revascularization, with pre-defined optimiszation criteria, was associated with improved clinical outcomes, when compared with angiographic guidance.^61^  *Table 3* outlines published optimization metrics through consensus^60^ and completed ICI studies.^12,57,61^

**Table 3:** Published optimization metrics for intracoronary imaging-guided bifurcation percutaneous coronary intervention

Optimization metric		de la Torre Hernandez^61^	LEMON OCT^12^	Bif-ARC^60^	DK-Crush VIII^57^
Stent expansion	MSA proximal	>90% prox. LM reference lumen^a^ *> 80% if tapering/funnelled LM* ^a^*if diffuse disease use 90%* *of smallest vessel area*	≥80% prox. LM reference lumen *Proximal segment defined to carina*	> 80% prox. LM ref vessel area	Absolute MSA criteria LM ≥10 mm^2^
	MSA distal	>90% prox. LAD/Cx ref. lumen	≥80% distal LAD reference lumen	> 80% distal branch ref vessel area	LAD ≥7 mm^2^ Cx ≥6 mm^2^ ≥ 90% distal reference lumen (defined as stent expansion index)
Landing zone	Plaque burden	<40% plaque burden	<40% plaque burden		<55% plaque burden
	Dissection	Dissection <45^°^ < 2 mm length	Dissection ≥60^°^ ≥ 2 mm length +/− medial extension	Dissection >60^°^ > 2 mm length	Dissection >3 mm length +/− medial extension
Stent deformation		Distortion or shortening of stent in longitudinal axis *− Recognize & correct*	Not defined		No stent fracture
Malapposition		≥0.5 mm > 2 mm longitudinal extent	≥ 0.4 mm ≥ 1 mm longitudinal extent	> 0.35 mm > 1 mm longitudinal extent	Not defined
Other stent metrics		Not defined	Tissue extrusion >500 µm		Stent symmetry index >0.8

Cx, circumflex artery; LAD, left anterior descending artery; LM, left main; MSA, minimal stent area.

^a^If diffuse disease use 90% of the smallest vessel area

Post-stent ICI should focus on a number of aspects of optimization: (i) stent expansion, (ii) landing zone—injury (dissection/haematoma) and geographical miss, (iii) stent deformation, (iv) SB patency (provisional approach), (v) malapposition, and (vi) eccentricity.

We will consider each of these elements in turn, but it is important to acknowledge that achieving all metrics of stent optimization, in every case, is challenging, as demonstrated in previous studies of ICI-guided PCI.^62^

### Stent expansion

Stent expansion requires quantitative assessment of the entire stent segment, including regions 5 mm proximal and distal to the stent. Automated lumen and vessel recognition facilitate real-time analysis in the routine clinical setting. Both absolute minimal stent area (MSA) values and relative expansion compared to reference segments should be assessed.

#### Absolute minimal stent area cutoff values

It is feasible to use absolute values in optimizing the LM, as its size varies less than any other coronary segment, however, gender and ethnic differences can impact the assessment. A gender difference in LM size has been confirmed by both CT and IVUS studies of disease-free populations, with larger dimensions demonstrated by IVUS [CT-derived LM diameters male 4.1 ± 0.6 mm vs female 3.3 ± 0.7 mm (*P* < .001)^63^ and IVUS-derived diameters male 4.3 ± 0.6 mm vs female 3.9 ± 0.5 mm (*P* < .001)^64^].

Recent trials recruiting Western patients have challenged the historical 5-6-7-8 criteria (Cx-LAD-bifurcation-LM lumen areas), defined in a Korean population, that predicted 9-month angiographic restenosis.^65^ IVUS subgroup analysis in EXCEL^66^ and NOBLE^26^ demonstrated MSA values within the LM of 9.9 ± 2.3 mm^2^ (*n* = 504) and 12.5 ± 3.0 mm^2^ (*n* = 224), respectively (*Table 4*). Consequently, the potential difference between populations from East and West has been debated. Interestingly, a very recent analysis of Korean patients undergoing upfront two-stent treatment of the LM has demonstrated a distal LM size comparable to the western data (10.9 ± 2.2 mm^2^).^67^ In this series, distal LM MSA was not predictive of cardiac events, whereas the distal outlet (branch) lumens demonstrated an association with 5-year major adverse cardiac events (MACE). The circumflex demonstrated the strongest association with an ostial MSA cut-off value of 5.7 mm^2^ (adjusted hazard ratio [HR] 2.77; *P* = .01). The ostial LAD MSA cutoff was 8.3 mm^2^ (adjusted HR 2.76; *P* = .03). Despite this, it is important to reflect that a significant proportion of patients fail to meet the absolute criteria (64.7%, 55.1% and 48.3% of patients had stent underexpansion in distal LM, ostial LAD and ostial Cx, respectively), in line with previous ICI studies defining optimization criteria.^62^

**Table 4:** Comparison of contemporary intracoronary imaging studies minimal stent areas achieved through image-guided bifurcation percutaneous coronary intervention

Study	Patient number	Stent strategy		Imaging modality		LM MSA (mm^2^)	LAD MSA (mm^2^)	Cx MSA (mm^2^)
		Provisional	2-stent	IVUS	OCT			
Kim *et al*. 2-stent crush^67^	292	0	292	292	0	10.9 ± 2.2	8.2 ± 1.7	5.9 ± 1.4
NOBLE IVUS substudy^26^	224	151	73	224	0	12.5 ± 3.0	10.1 ± 2.9	9.6 ± 3.4
EXCEL substudy^66^	290	-	-	290	0	9.9 ± 2.3^a^	-	-
LEMON OCT^12^	70	58	12	0	70	11.6 [9.6–14.3]	6.7 [5.7–8.3]	-

^a^EXCEL distinguished different post-PCI MSA values for distal bifurcation lesions (9.8 ± 2.3 mm^2^) vs ostial/shaft lesions (10.4 ± 2.5 mm^2^). Median post-PCI LM MSA 10.3 mm^2^.

We have provided an updated schematic of MSA targets for the segmented LM (dependent on LM crossover or two-stent technique) and have purposefully provided ranges to reinforce the need to consider these absolute values in relation to the reference segment vessel areas (*Figure 6*), as over-expansion of the left main to achieve target values carries significant risks and vessel perforation in this location has greater likelihood of mortality.

**Figure 6:**
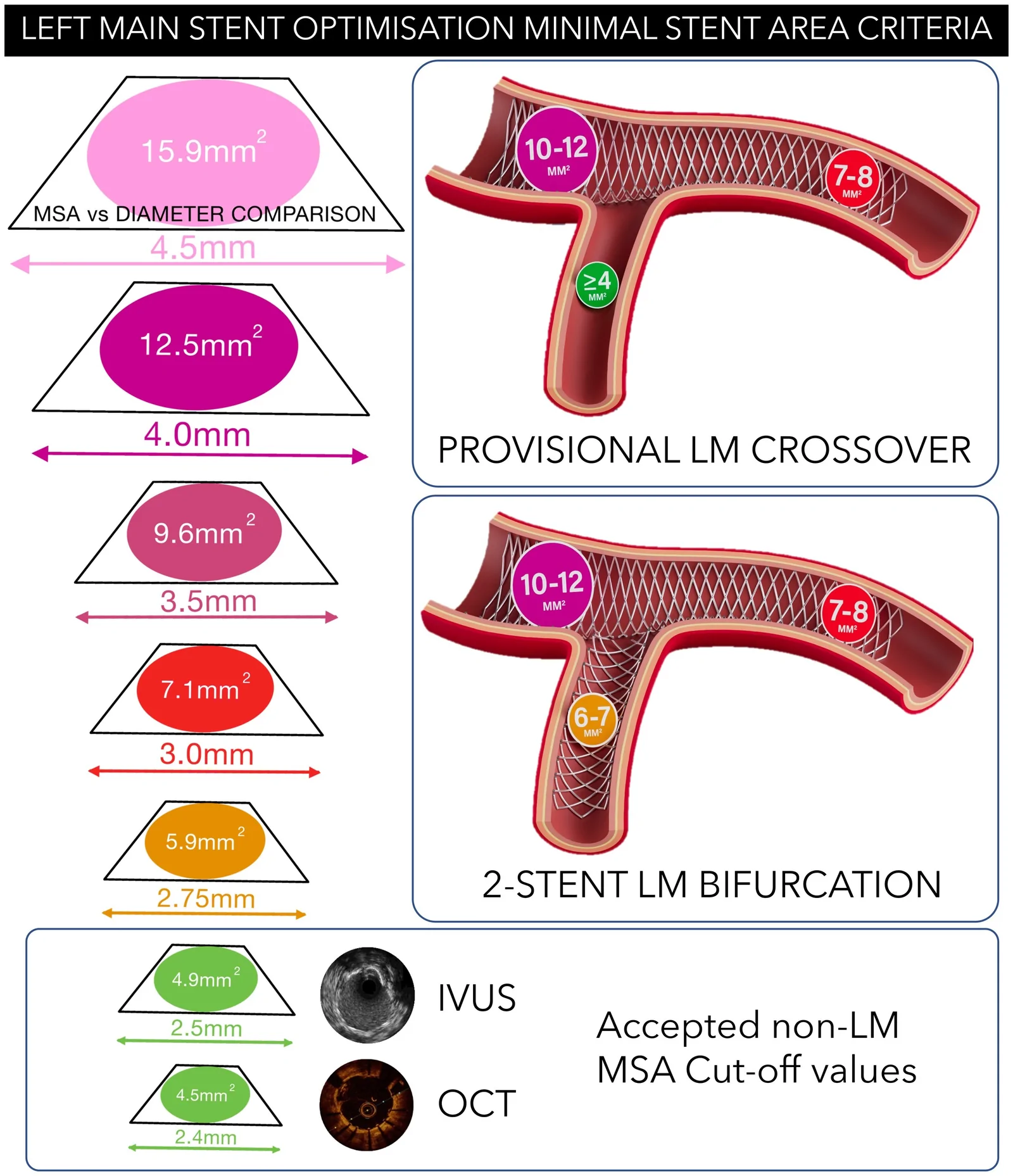
Stent optimization guidance for provisional LM crossover and 2-stent left main bifurcation PCI. Consideration of optimal stent areas and the equivalent lumen diameter is important to ensure that sizing of post-dilatation balloons is appropriate to the anatomy

#### Relative expansion indices

Concerns regarding the use of absolute MSA values, out of context with the underlying vessel size, led to the inclusion of relative expansion in the very first MUSIC trial IVUS criteria.^68^ Several formulas have been proposed to account for vessel tapering and calibre change between bifurcation vessel segments. In the LM, relative stent expansion could be calculated using the MSA and either the proximal or distal reference lumen area given the lack of SBs and consequently limited calibre change.

Contemporary studies of ICI-guided LM PCI have adopted 80–90% relative expansion as appropriate criteria for optimization (*Table 3*).^12,57,61^ However, it is important to acknowledge that diffuse disease or tapering of the LM segment may make defining the reference segment more challenging. De la Torre Hernandez and colleagues suggest that 90% of the smallest vessel area within the LM is used as reference where diffuse disease prevents assessment of the LM ostium. When treating a funnelled/tapering LM achieving stent expansion equivalent to 80% of the proximal vessel reference lumen is advised. Clearly, the need for blood clearance with OCT, and consequently the challenges posed if disease extends to the LM ostium, in addition to attenuation of OCT imaging in the presence of high burden lipid or necrotic core plaque, makes IVUS evaluation of the LM segment more reliable. These limitations of OCT have been acknowledged by the OCTOBER trial committee and they recommended tracing a circle using the available arc of external elastic membrane if visible between 60–170^°^. Where no healthy arc of vessel was visible they advised estimating the size according to Finet's law (D_LM_ = 2/3(D_LAD_ + D_Cx_)^34^), although these workarounds were only needed in a small fraction of cases.

Ultimately, both assessment of absolute MSA and relative expansion indices, across all components of the LM anatomy, provides the operator challenges in interpretation and may deter some from adopting an ICI-guided approach. Iterative improvements to both IVUS and OCT software have provided rapid and automated stent/lesion assessment, enhancing the time efficiency of procedural analysis. It is hoped that developments in ICI-derived physiological assessment will assist operators in optimization of PCI results,^69,70^ although the LM bifurcation will provide additional challenges in the application of these exciting technological developments.

### Landing zone—dissection management

The LM poses unique challenges when managing dissection because extending the stent proximally to ensure complete coverage may require substantial overlap or may result in unintended protrusion into the aorta. Consequently, where disease is extensive, on pre-PCI imaging, it is advised to stent to the ostium. In this situation, IVUS provides more consistent evaluation of the stent result due to an ability to withdraw the guide catheter back into the aortic root. However, where OCT has been used it is possible to use a guide-catheter extension (GCE) to provide visualization of the ostial vessel segment. Differences might exist between materials, and GCEs with limited metallic tip reinforcement appear to be the most suitable.^71^ Where disease has been identified to extend to the ostium, by angiography, we advise upfront selection of IVUS.

Where dissection is evident by imaging, with an arc extending >60^°^, extensive vessel injury, with extension into or beyond the media and a longitudinal extension of >3 mm, the placement of an additional stent is likely to be appropriate.

### Stent deformation

Complex bifurcation PCI inevitably involves multiple steps and the delivery of numerous devices through freshly deployed stents. It has long been acknowledged that device interaction, particularly the guide-catheter or tip of a balloon catheter can result in deformation of the stent architecture^72^ with loss of integrity of vessel scaffolding, non-uniform delivery of anti-restenotic drug and stent protrusion/malapposition that may increase the risk of subsequent stent thrombosis.

Recent data provided from a pre-specified analysis of the OCTOBER study has identified unintended stent deformation (USD) as an important finding in post-stenting OCT evaluation.^5^ It was observed in 9.3% of all OCT-guided cases, with a greater incidence in LM bifurcation PCI (18.5%), predominantly caused by abluminal wiring or catheter collision (*Figure 7*). Unfortunately, when compared to corelab, clinical sites failed to recognize or treat the stent deformation in 55% of cases, with an associated MACE rate of 23.3%. However, where stent deformation was recognized and treated during the index procedure no MACE was reported.

**Figure 7:**
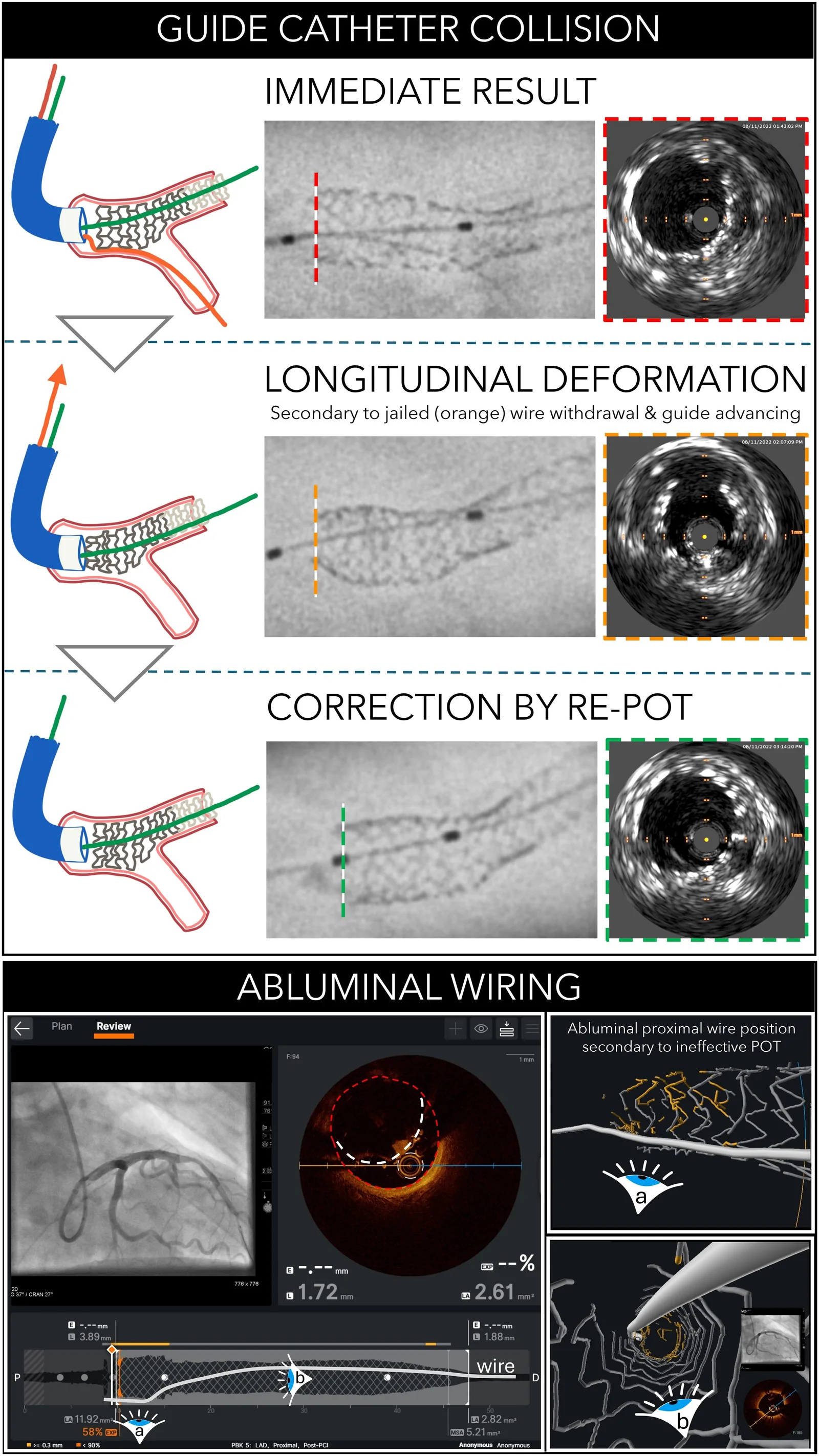
Examples of unintended stent deformation detected by IVUS & OCT. GUIDE COLLISION—The proximal optimization technique (POT) ensures appropriate expansion and apposition of the left main stent segment, confirmed by angiographic stent optimization software and IVUS (red-dashed outline). However, withdrawal of the jailed guide wire (orange line) resulted in guide catheter collision and proximal stent distortion (strut malapposition and longitudinal shortening), confirmed by stent optimization software and IVUS (orange-dashed outline). Repeat POT corrected the distortion, confirmed by stent optimization software and IVUS (green-dashed outline). ABLUMINAL WIRING—Cross-sectional OCT imaging identifies the abluminal position of the catheter and wire in a segment of left main malapposition (lumen contour highlighted by red line & stent struts by white dashed line, with automated OCT software identifying strut malapposition >0.3 mm (yellow bar and 3D-strut reconstruction). 3D reconstruction with removal of tissue facilitates detection of the wire position in relation to stent contour, most evident viewing from outside the stent (viewpoint a) & from distal to proximal within the stent lumen (viewpoint b).

Operators must be mindful of the risk of stent-device interaction, particularly the guide-catheter, the tip of a balloon catheter or the imaging probe can result in stent deformation, with disruption of vessel scaffolding, non-uniform delivery of anti-restenotic drug and stent protrusion/malapposition that may increase the risk of subsequent stent thrombosis. Clearly, the POT was developed,^73^ and has been consistently endorsed by the EBC,^74^ to minimize the risk of stent deformation. Furthermore, we have supported the concomitant use of stent enhancement imaging to provide useful information regarding stent deformation,^75^ and this should be considered as an adjunct to ICI evaluation. Real-world registry data have demonstrated that intravascular image-guided POT provides better outcomes,^76^ with independent prediction of target lesion failure (adjusted HR 0.65, 95% confidence interval 0.48–0.87; *P* = .004). Despite OCT-guided POT in 99% of cases, USD was still observed in OCTOBER indicating that abluminal rewiring still occurs despite POT and occurs in other segments, primarily the ostial part of a SB stent. Other factors, such as guide catheter collision due to a jailed wire and collision with balloon and guide-extension catheters are critical procedural steps associated with considerable risk of USD (*Figure 7*).

Beyond abluminal wiring and guide-catheter collision, recent bench simulation and clinical reports have highlighted novel mechanisms of POT-related stent elongation^77,78^ and despite mandated use of POT in all bifurcation PCI, continued attention to avoid stent injury is necessary.

ICI and in particular OCT can be effectively used for evaluating wire positions to avoid accidental abluminal rewiring reducing the risk of USD (*Figure 7*). We strongly endorse that ICI evaluation of the LM PCI result is the last procedural step, to confirm that optimization has been achieved and to ensure avoidance of USD.

### Side-branch optimization

#### Left main-crossover (provisional) PCI

We have already considered predictors of circumflex compromise in 1.4.5, however, when the circumflex appears angiographically sub-optimal post-crossover stenting, it may be necessary to optimize both branches through repeated POT, final kissing balloon inflation and possible LM post-dilatation as residual circumflex stenoses >70% are associated with increased hazard of death and myocardial infarction.^79^

Angiographic evaluation overestimates the functional severity of jailed SB lesions with several studies demonstrating a lack of correlation between FFR and percent diameter stenosis in the ostium of the SB. Physiological assessment of the circumflex after crossover stenting of the LM has been proposed as a way to evaluate the real impact of angiographic stenosis observed in the SB after main vessel stenting. A study performed in 83 patients demonstrated that those with FFR <0.80 in the jailed LCx after cross over LM-LAD stenting had a higher rate of target lesion failure at 5-year follow-up, while there was no correlation between angiographic percent diameter stenosis and events.^80^ Physiological assessment of the jailed LCx may be appropriate after LM stenting to evaluate the need of further interventions.

A post-PCI circumflex MLA <3.9 mm^2^ has been shown to predict functional SB compromise (FFR <0.8).^81^ ICI evaluation of the SB requires clearance of the main vessel stent struts from across the SB ostium and in a large vessel this is best achieved by kissing-balloon inflation. Following LM crossover stenting, a distal rewiring of the circumflex is preferred, however, the OPTIMUM 3D-OFDI study demonstrated that optimal rewiring is only achieved in 55.4% of cases on first attempt, with a median number of OFDI runs of 1 [interquartile range 1–3] to achieve optimal position.^82^ Although OFDI guidance led to reduced incomplete stent apposition and long-term neointimal healing, a clinical benefit has not been established. The OCTOBER protocol acknowledged the potential for suboptimal wiring and mandated OCT assessment following wire recross, however, outcome data supporting optimization of wire position in a jailed SB ostium have not yet been presented. Similar to the findings of USD, it must be acknowledged that ICI interpretation for this purpose is challenging. Consequently, the use of ICI to guide wire recross and subsequent optimization has not been widely adopted. Advances in 3D reconstruction of ICI and increased operator experience may impact practice in the future.

#### Two-stent strategies

Two stent approaches to the treatment of complex bifurcation lesions require many more technical steps and are likely to increase the risk of USD. Consequently, ICI may play a more important role in guiding peri-procedural steps in complex treatment. However, operator fatigue, challenges of ICI interpretation, as discussed in Section *Stent deformation*, plus an absence of definitive outcome data limit our support for the implementation of additional ICI assessment of either SB or main vessel during PCI. If complications or difficulty in the delivery of devices are encountered during the procedure, we would strongly endorse the use of ICI to clarify the situation, specifically focusing on wire position and evidence of stent deformation.

The circumflex ostium is particularly vulnerable to stent failure and consequently ICI evaluation following deployment of a two-stent strategy is very important.^83^ Ideally, stent underexpansion is avoided through appropriate pre-PCI lesion assessment but frequently repeated postdilatation, following stenting, is required to achieve an acceptable MSA (circumflex MSA 6–7 mm^2^) (*Figure 6*).

### Malapposition

Malapposed and uncovered stent struts have been frequently observed in the setting of stent thrombosis, particularly in association with stent underexpansion.^84^ Although small degrees of malapposition, and specifically strut coverage of an SB, have often been considered an ‘innocent bystander’, a novel pathological study of LM stent failure demonstrated these features to be the strongest predictors of pathological stent failure.^85^ However, this post-mortem study was unable to distinguish acute and late acquired malapposition. Accordingly, in one of the largest observational OCT studies including 1290 patients, followed for at least 3 years, significant malapposition, defined as a total malapposition volume ≥7 mm^3^, was strongly associated with ischemic events (cardiac death, target-vessel related myocardial infarction and stent thrombosis).^86^ In contrast, other studies including the ILUMIEN IV RCT have failed to demonstrate a relationship between acute stent malapposition and clinical events.^87^ Collectively, this consensus group suggests that minor malapposed segments need not be corrected (<400 μm) but larger segments of malapposition (>400 μm with longitudinal extent >1 mm) should be corrected where anatomically feasible and safe. Furthermore, it is advised to reassess reference segment vessel dimensions on post-PCI ICI evaluation and where positive vessel adaptation exceeds the original stent diameter selection, an appropriately sized post-dilatation balloon should be used to overcome stent undersizing and associated malapposition.

Clearance of ostial SB struts is more controversial, and although the EBC recommends the provisional approach incorporates POT, distal SB rewiring, followed by kissing balloon inflation,^56^ randomized data confirming enhanced clinical outcomes are lacking. However, potential advantages may include facilitated access to lesions originating in the distal vessel regions. In the absence of ICI it is impossible to confirm effective stent apposition, wire crossing and ultimately stent optimization, and consequently such manoeuvres may jeopardize rather than enhance the acute stent result. The OCTOBER study was the first RCT to mandate the use of ICI in confirming each step of the bifurcation PCI procedure, so we await further analysis to assess if OCT can enhance the acute procedural outcomes with regards malapposition and ostial strut clearance.

### Stent eccentricity

Having undertaken final kissing balloon inflation it has been shown on bench that extension of the distal main vessel and SB balloons back into the proximal main vessel may result in stent eccentricity and proximal malapposition.^88^ Stent eccentricity is calculated by dividing the minimum and maximum stent diameters in individual cross-sections of the stent and deemed significant if <0.80.

A small study has associated stent eccentricity with uneven healing (neointima) and postulated that this may be a key mechanism for subsequent stent thrombosis.^89^ Despite uncertainty regarding the relevance of stent eccentricity, it should be noted that the DK-Crush study group have included a stent symmetry index >0.8 as an imaging criteria for optimization in DK-Crush VIII.^57^

## Conclusions

A growing body of evidence supports the use of ICI to guide complex PCI. LM disease has been recognized as a specific lesion-subset that benefits from an image-guided approach. The intention of this document is to outline best practices in the pre- and post-PCI evaluation of LM intervention (*Figure 8*).

**Figure 8:**
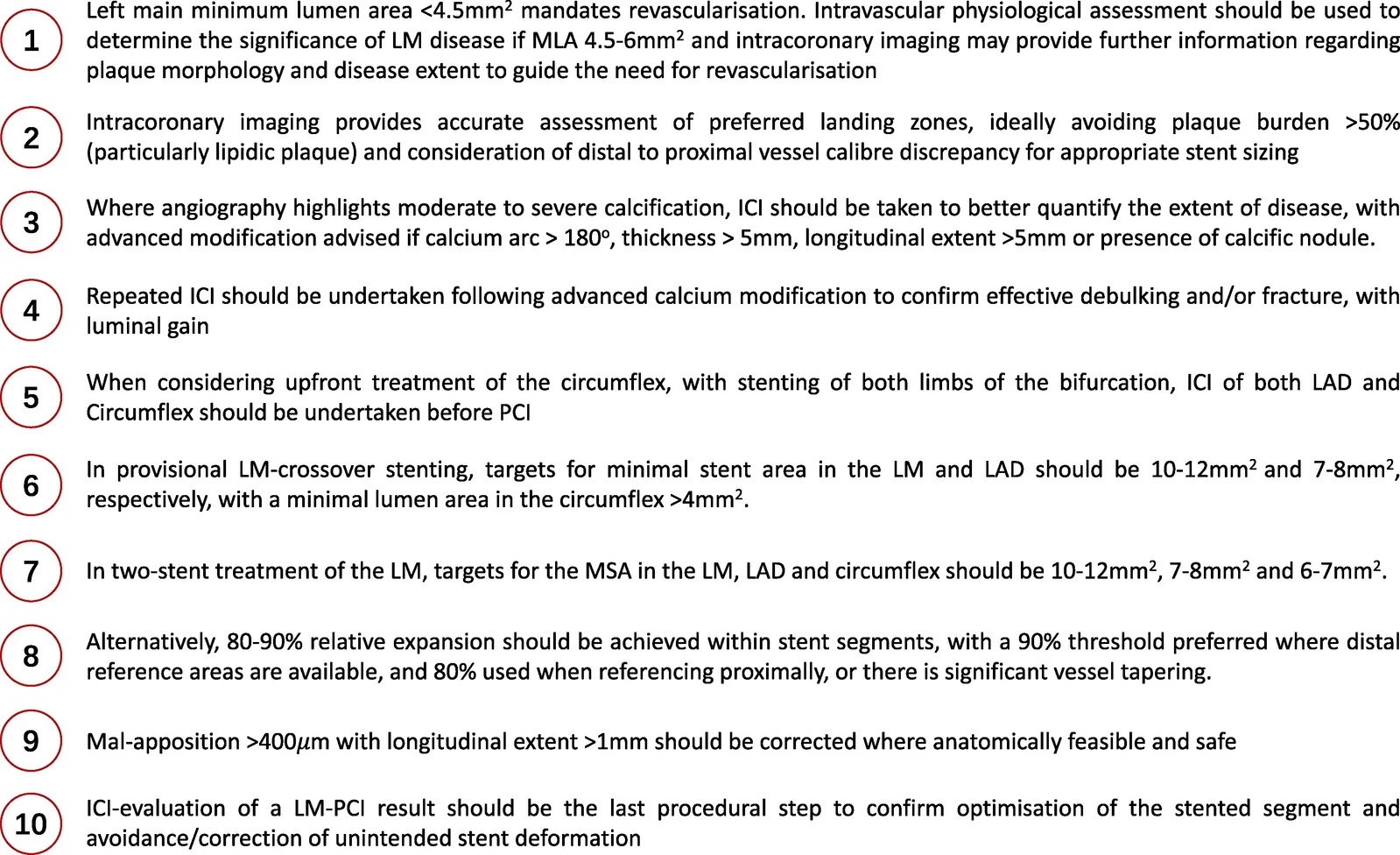
Top 10 Key learning points from the Intracoronary Imaging for left main PCI consensus statement

The recent American College of Cardiology and European Society of Cardiology guidelines recommendations require the interventional community to adapt their practice and embed ICI into their PCI decision-making and procedural optimization. Challenges exist to ensure patients gain access to these best practices, through changes to reimbursement, education of the interventional community and further iterative improvements in the technology. Most importantly, it must be recognized that ICI provides procedural support pre- and post-stenting, but should not be perceived as a simple accessory to angiographically-guided intervention. Effective ICI adoption requires a standardization of approach, ensuring high-quality execution, greater homogeneity of results between operators and across centres, with an aspiration for more predictable and durable long-term outcomes for patients. To achieve this, operators must be prepared to adapt their practice to the additional information provided by ICI and be both willing and humble to defer, adapt and/or react to information provided by these exciting technologies.

## Declarations

### Data Availability

No data were generated or analysed for or in support of this paper.
